# Surgical treatment of long-standing overt ventriculomegaly in adults (LOVA)

**DOI:** 10.1007/s00701-016-2998-7

**Published:** 2016-11-02

**Authors:** Ronak Ved, Paul Leach, Chirag Patel

**Affiliations:** University Hospital of Wales, Cardiff, CF14 4XW UK

**Keywords:** Long-standing, Overt, Ventriculomegaly, Adults, Hydrocephalus

## Abstract

**Background:**

Longstanding overt ventriculomegaly in adults (LOVA) is characterised by chronic hydrocephalus presumed to begin during infancy, but arresting before becoming clinically detectable. Later in life clinical features of hydrocephalus ensue, typically in the 5th or 6th decades. Only a relatively small number of LOVA case series have been published, and ambiguity remains regarding optimal management. This case series describes a series of patients with LOVA treated successfully at a single neurosurgical institution using endoscopic third ventriculostomy (ETV).

**Methods:**

A series of 14 patients were diagnosed with LOVA using established clinical and radiological criteria. All patients underwent an ETV and their clinical conditions were followed up for up to 5 years post-operatively.

**Results:**

Fourteen patients (100 %) reported either improvement or halt of progression in their presenting symptoms 3 months after ETV; 93 % of patients (*n* = 13) did not require any further surgical intervention. One patient (7 %) reported deterioration in symptoms beyond 3 months post-operatively, which necessitated further surgery (ventriculoperitoneal shunt). These promising outcomes after ETV are mirrored in numerous other LOVA case series. Other works have analysed the value of CSF shunting procedures in LOVA, with mixed results. A direct, prospective comparison of outcomes after shunt procedures and ETV, with a specific focus on LOVA patients, is yet to be completed. A minority of patients fail to respond, or develop recurrence of symptoms, months or years after initial surgical intervention.

**Conclusions:**

ETV is an attractive option for surgical treatment of LOVA. After surgical treatment for LOVA, long-term follow-up should be considered to screen for late recurrence of the condition.

## Introduction

The term “LOVA” was first used by Oi and colleagues [1] to define a cohort of adult patients with symptoms of chronic hydrocephalus, a head circumference of more than 2 standard deviations above the 98th percentile and overt tri-ventriculomegaly on neuroimaging, in the absence of a secondary cause for aqueductal stenosis in adulthood. The mechanism for this phenomenon remains unclear [2]. It is hypothesised that there is a full or partial obstruction of CSF flow through the aqueduct of Sylvius before fusion of cranial sutures (i.e. before age 24 months) followed by restoration of CSF flow before clinical symptoms can manifest in childhood. This theory explains the large head circumference and initial asymptomatic period of patients with true LOVA. This restoration in CSF may be explained by re-establishment of flow through the aqueduct, use of alternative flow pathways, modification of CSF production or a combination of these mechanisms [1, 3].

**Fig. 1:**
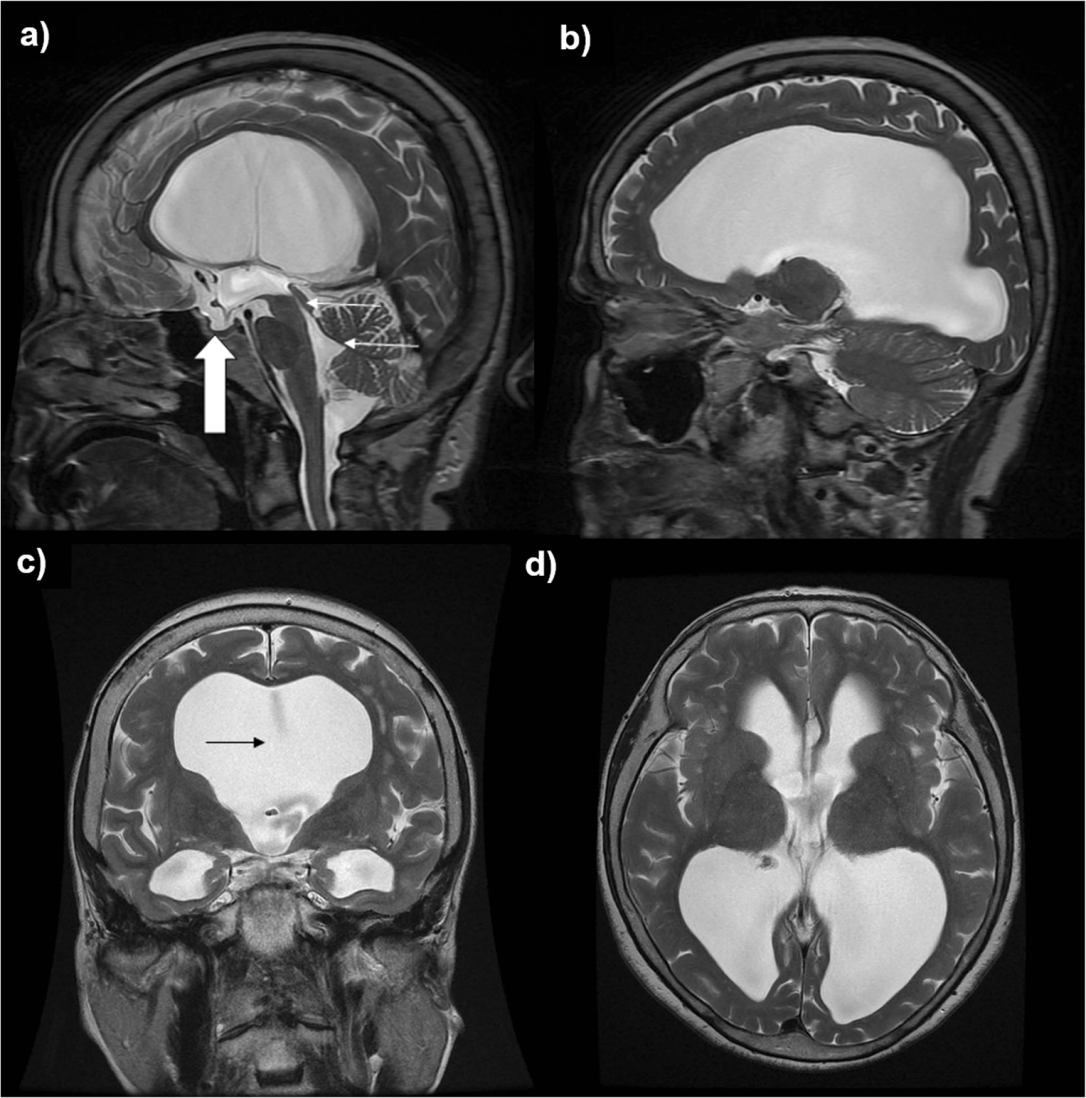
T2-weighted magnetic resonance images from a patient with LOVA: Case 14 in our case series. **a** and **b** Sagittal slices demonstrating triventriculomegaly, with sparing of the cerebral aqueduct and fourth ventricle (*thin white arrows*). There is apparent enlargement of the sella turcica due to the chronic nature of the hydrocephalus (*thick white arrow*). **c** Coronal slice demonstrating obliteration of the septum pellucidum (*black arrow*). The triventriculomegaly can once again be identified. **d** Subtle sulcal effacement may be appreciated, particularly in the right temporo-parietal region, on this axial slice from the same scan. However, this patient’s age-related (81) cerebral atrophy renders this effacement less marked than in typical cases of LOVA (where presentation is in the 5th–6th decades)

Later in adulthood, ill-defined alterations in CSF flow dynamics lead to a return of the previously arrested hydrocephalus and thus the gradual onset of symptoms of hydrocephalus, such as headaches, cognitive decline^4^, imbalance, visual problems and psychological disturbance [4]. Neuroimaging typically reveals triventricular hydrocephalus with sparing of the fourth ventricle and absence of aqueductal stenosis. Exemplar T2-weighted magnetic resonance images of a patient with LOVA are given in Fig. 1.

The process is typically slow and progressive in nature, and a loss in brain parenchyma plasticity makes treatment outcomes of LOVA difficult to predict. There are conflicting case series describing outcomes for patients with LOVA after neuroendoscopic and shunt-based CSF diversion procedures, with each series advocating different opinions as to the optimal management for this debilitating condition.

This case series collates outcome data from LOVA patients treated using endoscopic third ventriculostomy (ETV) at our institution and reviews current data published about the proposed pathophysiology, diagnosis, treatment options and patient outcomes for LOVA.

## Methods

Fourteen patients were diagnosed with LOVA at a single neurosurgical unit between 2011 and 2016, by virtue of all the patients meeting the clinical and radiological criteria for LOVA established by Oi et al. [2] (Table 1). All patients underwent an ETV utilising the same standardised operative technique, outlined by Al-Jumaily et al. [4]. A single burr hole was performed in the midpupillary line just anterior to the coronal suture. Disposable rigid endoscopes were used to create a stoma in the floor of the third ventricle using a figure-of-eight balloon. If present, any secondary membrane in the pre-pontine cistern was opened.

**Table 1:** Clinical and radiological criteria used to confirm the diagnosis of LOVA in the presented case series (adapted from Oi et al., 2000 [1])

1. Clinical symptoms of hydrocephalus developing in adulthood—e.g. headaches, cognitive decline, imbalance, gait disturbance, psychological disturbance, visual deterioration/diplopia
2. Macrocephaly defined by head circumference >98th percentile in adulthood (male 53.8 cm; female 52.9 cm) [5, 14]
3. Overt tri-ventriculomegaly (lateral and third ventricles) on neuroimaging, with cortical sulcal effacement and/or destruction of the sella turcica as evidence of long-standing ventriculomegaly
4. Absence of a secondary cause for aqueductal stenosis in adulthood (e.g. previous meningitis, subarachnoid haemorrhage)

Outcomes were assessed by clinical review of patients in outpatient clinics (minimum 6 months, maximum 5 years) post-operatively. If discharged from clinic, patients and their primary care physicians were asked to contact the neurosurgical unit if symptoms recurred [6].

MEDLINE® database searches were conducted to retrieve articles related to LOVA. Search terms included “long-standing overt ventrioculomegaly in adults”, “LOVA”, “adult hydrocephalus”, “adult ventriculomegaly” and “arrested hydrocephalus”. Two reviewers independently scrutinised each retrieved article, evaluating the relevance of the study’s sources and analysed the main findings of each study. A summary of the results from the studies deemed relevant for analysis is outlined in Table 2.

**Table 2:** Summary of MEDLINE® results for published case series of LOVA patients treated with CSF diversion, via shunt or ETV

Authors (year)	N_o._ of LOVA patients (total in cohort)	Primary interventions (n)	Outcomes	Complications	Caveats
Oi et al. (2000) [1]	18 (18)	ETV (8); VP shunt (9) (DPV 7; PPV 2)	Clinical improvement and radiological arrest of hydrocephalus in all patients after primary ETV and PPV	SDH in all 7 patients receiving DPV shunts, requiring shunt revision to PPV or ETV Two intra-axial haemorrhages after ETV. One case of transient visual field deficit after ETV	Relatively small cohort
Keifer et al. (2002)	23 (23)	Gravitational-shunt (23)	82 % (19) patients reporting symptomatic improvement	2 SDH—one necessitating operative drainage	Non-comparative study; potential selection bias; relatively small cohort
Keifer et al. (Jan 2005)	30 (30)	Gravitational shunt (30)	87 % (26) patients reported improvement in pre-operative symptoms	2 post-operative hygromas—one requiring shunt revision; limited or transient improvement in pre-operative symptoms in 3 (10 %) patients	Non-comparative study; follow-up of up to 12 months only, therefore late shunt infections or failures potentially not captured; potential selection bias; relatively small cohort
Keifer et al. (July 2005)	26 (26)	Gravitational shunt (26)	87 % (22) of patients reporting clinical improvement	2 SDH; 4 cases with symptoms of over-drainage, 2 requiring shunt revision; one case of severe weight gain (due to underdrainage) requiring replacement of the shuntAssistant® portion of the shunt; one case with recurrence of symptoms	Non-comparative study; patient selection potentially affected by suboptimal ETV equipment; relatively small cohort
Canu et al. (2005) [7]	1 (1)	N/A	Identification of preserved language and praxis functions despite severe ventriculomegaly	N/A	Single case report; no therapeutic interventions performed
Rekate H. (2007) [13]	6 (6)	ETV (6)	CSF flow through ETV confirmed radiologically in all cases post-operatively	Persistent symptoms in 5 cases (83 %) necessitating VP shunt or venous stenting. Mild short-term memory deficits in 1 patient	Small cohort; heterogeneous patient population, including three (50 %) patients <30 and one <20 (age 16). ETVs within the study cohort performed at two different institutions
Hamanda et al. (2009) [8]	1 (1)	ETV (1)	Improvement in headaches, memory, cognitive and constructional abilities	None	Single case report; patient had a history of operated myeloschisis and aqueductal stenosis as a child (potentially not a true case of LOVA)
Jenkinson et al. (2009) [6]	24 (190)	ETV (24)	88 % (21) reporting improved symptoms after ETV	9 post-ETV complications in the total cohort (5 %): 2 minor SDHs; 2 transient focal neurological deficits; 2 CSF leaks; 3 ICHs (2 necessitating EVD placement)	Heterogeneity of indications for ETV; small numbers of patients in each subgroup; outcomes defined by clinical assessment in outpatient clinic
Al Jumaily et al. (2012) [4]	20 (20)	ETV (20)	Improvement in headache (18; 90 %); improved balance (12; 80 %)	Persistent headaches in 2 patients (10 %), requiring repeat ETV and gravitational shunt insertion, with persistence of symptoms despite shunt insertion in one patient. Two short seizures immediately post-operatively; poor cognitive performances persisted across the cohort post-ETV	Patients failing to respond to ETV may have been suffering from non-ICP-related chronic daily headaches—however ICP monitoring was not performed to confirm this
Ono et al. (2012) [10]	1 (1)	Pressure programmable valve VP shunt (1)	Improvement in memory	SDH requiring drainage and second shunt insertion	Single case report
Issacs et al. (2016)	97 (163)	ETV (163)	130 (87 %) of total patient cohort reported improvement in symptoms at 3 months post-ETV	10 (6 %): meningitis (4); SDH (2); focal neurological deficit (1); memory deficit (1); weight gain (2). No long-term disability	Data not specific to LOVA (cohort includes ETVs for failed VP shunts, secondary hydrocephalus and NPH)
Ved et al. (2016)	14 (14)	ETV (14)	14 patients (100 %) reported improvement or halt of progression in presenting symptoms 3 months post-operatively; 97 % (13)	One (7 %) post-operative seizure with no long-term disability; one (7 %) patient requiring a second procedure (VP shunt) due to symptom recurrence	Relatively small cohort. Outcomes defined by clinical assessment in outpatient clinic

SDH = subdural haematoma; NPH = normal pressure hydrocephalus; ICH = intracerebral haemorrhage

## Results

Table 3 depicts the demographics, presenting symptoms and outcomes for the 14 cases of LOVA treated at our institution from 2011 to 2016. There were eight males and six females, with a mean age of 51 (22–81). All 14 patients met the diagnostic criteria outlined in Table 1. All patients underwent ETV in the first instance. Follow-up ranged from 6 to 60 months (median 36 months). The mean head circumference was 58.2 cm.

**Table 3:** Case series of 14 patients diagnosed with LOVA and treated with primary ETV. Outcomes were reviewed at 3 months and subsequently between 6 months–5 years post-operatively

Case number	Sex	Presenting symptoms	Age at presentation	Head circumference	Operative complications	Outcome at 3 months	Outcome beyond 3 months
1	M	Leg weakness, falls, Headaches	43	55 cm	No	Reduced headache frequency, “80 % better”	5 years: no deterioration; “100 % better”
2	M	Unsteadiness	38	58 cm	No	No deterioration in balance	5 years: no deterioration
3	M	Dizzy spells, mood swings and headaches	53	62 cm	No	Improved memory and headaches; no deterioration in mood swings	5 years: mild headaches and dizziness returned
4	F	Poor mobility and headaches	68	59 cm	No	Improved mobility and headaches absent	5 years: improved mobility and headaches
5	M	Ataxia, seizures, and poor memory	34	54 cm	Yes (Generalised tonic clonic seizure)	Improved ataxia and seizure frequency; no deterioration of memory	5 years: no further deterioration
6	F	Poor mobility and headaches	57	59 cm	No	Improved mobility and headaches	4 years: return of headaches; awaiting outpatient review
7	M	Unsteadiness	63	60 cm	No	Balance improved	3 years: “balance back to normal”
8	M	Diplopia	59	60 cm	No	Diplopia resolved	36 months: return of diplopia and onset of leg weakness: VP shunt inserted
9	F	Imbalance	75	57 cm	No	Improved balance and mobility	2 years: no further deterioration
10	M	Headaches and imbalance	22	58 cm	No	Improved headaches and balance	2 years: balance further improved
11	M	Headaches and cognitive decline	55	59 cm	No	Improved headaches and cognition	1 year: cognition further improved
12	F	Headaches	41	59 cm	No	Improved headaches	1 year: no further deterioration
13	F	Headaches	17	57 cm	No	Headaches absent	1 year: no further deterioration
14	F	Memory disturbance, disinhibition, and unsteadiness	81	58 cm	No	Socially and cognitively much improved at 4 months	6 months: continued cognitive and social improvement

One patient (7 %) experienced a post-operative complication after ETV in the form of a single tonic-clonic seizure immediately post-operatively; there were no other complications post-ETV. All 14 patients (100 %) reported either improvement or halt of progression in their presenting symptoms 3 months after ETV; 93 % of patients (*n* = 13) did not require any further surgical intervention. Only one patient (7 %) reported deterioration in symptoms beyond 3 months that necessitated further surgery (VP shunt). Two patients have recently reported minor headaches at 4 and 5 years follow-up post-operatively and are awaiting review from the senior author.

## Discussion

### Demographics

The clinical onset of LOVA can occur at any stage in adulthood, with a range between 22 and 81 years (median 54) in our case series, which has been mirrored by others [4, 7, 8]. No clear-cut differences in patient sex have been identified thus far. Some series have identified a trend towards sub-normal IQ and cognitive abilities in LOVA patients, although this feature does not appear to be universal across all cases of LOVA [4, 7, 8]. Given the progressive nature of LOVA, early identification of patients with potential symptoms and signs of the condition is an attractive notion, as it may help to maximise the impact of any surgical intervention to implement it earlier in the disease process. However, the subtle myriad of early symptoms and the non-specific nature of large head circumference make early detection of LOVA a clinical challenge.

### Pathophysiology

The key features of LOVA are aqueductal stenosis, with arrest of hydrocephalus before the onset of gross macrocephaly and the symptoms of raised ICP. During the arrested hydrocephalic period CSF flow is likely maintained via a combination of rencannalisation of the aqueduct, utilisation of alternative CSF flow routes, and modification of CSF production or absorption [1]. This asymptomatic interval is followed later in life by a failure of the compensatory processes, leading to the symptomatic phase of LOVA, which is typically progressive and long term. Patients with an identifiable insult to alter CSF flow are excluded from the definition of true LOVA, and the mechanisms that alter CSF dynamics to end the asymptomatic phase of the condition remain unclear.

It is thought that the symptoms of LOVA develop as a consequence of (1) failure of adequate CSF flow, (2) skull base changes as a consequence of chronic and progressive rise in ICP and (3) chronic pressure effects upon brain parenchyma [1, 9, 10]. As such, early intervention has the potential to modify the disease process before irreversible changes occur as a result of chronically raised ICP [4].

Reports of persistent symptoms in patients with LOVA after both ETV and shunt procedures have led to a theory that, in some cases, the pathological process in adulthood leading to CSF volume imbalance may actually take place more distally than the aqueduct; it may be a phenomenon of failure of CSF re-absorption, thus unnameable to traditional CSF diversion procedures. One case reported by Rekate [13] illustrated a LOVA patient whose symptoms remained refractory to CSF shunt diversion and ETV, which in itself was complicated by shunt infection. A venogram revealed bilateral transverse sinus stenosis, which was successfully treated utilising a neuro-radiological venous-stenting procedure. The patient’s ICP subsequently normalised. It is possible that back-pressure from blockade of dural venous sinuses could instigate a chronic, potentially reversible, aqueductal stenosis.

### Symptomatology

Al-Jumaily et al. identified the most common features in LOVA were headaches and imbalance [4], which were also the most common presenting symptoms in our case series (Table 3).

The same group also highlighted that patients with LOVA may demonstrate a myriad of cognitive and psychological problems such as decline in memory, attention and language skills, along with depression, anxiety and disinhibition [4, 7, 8]. Some case series have retrospectively identified that a proportion of LOVA patients demonstrated sub-normal IQs or evidence of early dementia before the onset of more overt symptoms of raised ICP, such as headaches and gait disturbance [4, 7, 8]. However other patients demonstrate normal-to-high IQs and report no evidence of hydrocephalic symptoms at childhood, with a large head circumference the only pre-symptomatic indicator of a potential CSF flow problem [3, 4]. Whether any pre-symptomatic decline in cognitive function identified in a proportion of LOVA patients is a consequence of the arrested hydrocephalus at childhood or whether it represents the earliest phase of the decompensation of CSF flow in adulthood remains to be explored.

These symptoms may be difficult to identify clinically, as the chronic nature of LOVA may permit some patients’ brains undergo sufficient neuroplasticity to permit functional reorganisation, minimising any clinical neuropsychological deficits until late in the disease process [4, 8]. Nevertheless, when cognitive decline, inattention and mood disturbances do occur in LOVA, they can have significant impacts upon quality of life [4]. These symptoms were marked in one case from the cohort at our institution. In this case there was significant improvement in the social functioning, memory and mood after ETV (Table 3). Whilst there are other reports of improvement in these faculties after surgical treatment of LOVA [8], it is thought that these symptoms are less likely to improve if treatment is implemented late in the disease process [4]. Neuropsychological symptoms should therefore be assessed and considered early in the therapeutic decision-making process for LOVA patients who may appear to function highly even after the point of clinical decompensation ensues [4, 6–8].

### Treatment

All reviewed studies advocated surgery for symptomatic cases of LOVA. However, the optimal CSF diversion procedure for patients with LOVA has been debated for over a decade [1, 2, 9–12]. This debate has centred on a debate between neuroendoscopic procedures versus CSF shunt diversion. Oi et al. [1] reported outcomes for 18 patients with LOVA, 9 undergoing neuroendoscopic ETV (8) or aqueduct-plasty (1) and 9 receiving ventriculoperitoneal (VP) shunts [7 differential pressure valve (DPV) shunts, 2 pressure programmable valve (PPV) shunts]. All seven patients receiving DPV shunts developed subdural haematomas, most likely as a consequence of over-drainage. All of these patients required shunt revision to a PPV shunt and/or ETV. Six of the nine patients undergoing neuroendoscopic procedures developed radiological and clinical arrest of hydrocephalus. Two patients in this study suffered intra-axial haemorrhages after ETV. However, subsequent series have demonstrated the relative safety of ETV, with an overall complication risk of approximately 6 % [4, 13]. In one case series, a single ETV provided long-term symptomatic relief for 88 % (21/24) of patients with LOVA with no long-term complications port-operatively [6]. This correlates with our series, where there was only one complication post ETV: a self-limiting generalised seizure with no long-term sequelae for the patient. Conversely 100 % (*n* = 14) of patients in our cohort reported improvement or absence of progression in their symptoms at their first follow-up appointment after ETV (3 months).

There are no case reports of significant change in ventricular diameter after ETV or shunt insertion. The explanation touted for this is that only a small volume of CSF needs to be drained to achieve near-physiological ICP in adult hydrocephalus [11]. Improvements in radiological CSF flow and ICP after ETV for LOVA have been identified [1, 13]. However, these do not appear to correlate with clinical improvement, and recurrence of symptoms does not reliably relate to failure of flow through the ETV stoma [13]. It seems that after initial drainage of CSF through the stoma, a more complicated reorganisation of CSF dynamics takes place. Unpacking this mechanism may then be the key to understanding how to achieve sustained relief from the symptoms of LOVA.

The majority of studies, our series included, demonstrate that a majority of LOVA patients can achieve post-operative clinical improvement in headaches, balance, motor skills and neuropsychological function after ETV [3, 4, 7, 8]. One large retrospective study of ETV in adult patients (*n* = 163) recently identified that the majority of patients undergoing primary ETV (87 %; *n* = 130) reported subjective improvement in symptoms 3 months post-operatively [3]. Only ten patients (6 %) suffered post-operative complications. It should be noted that the authors present data relating to their cohort as a whole, which encompassed adult patients with normal pressure hydrocephalus, partially treated childhood hydrocephalus (i.e. failed VP shunt), hydrocephalus secondary to other intracranial pathologies and LOVA. Separate outcome data for these specific causes of adult hydrocephalus were not presented; however, 97 patients in the whole cohort (60 %) did meet the diagnostic criteria for LOVA, and their outcomes for ETV success and complications align with data from other case series specifically reviewing cases of LOVA (Table 2).

Despite these impressive outcomes after ETV, other works report less promising results. In one series, six patients with LOVA underwent ETV [10]. All six patients required a secondary procedure for persistent symptoms: shunt insertion, repeat ETV or venous stenting. This small series highlighted the need for continued follow-up whatever intervention is implemented, as 50 % of patients (*n* = 3) suffered recurrence of symptoms at 18–36 months after their primary ETV, despite an initial improvement or arrest in progression of symptoms. Other studies subsequently identified varying long-term success rates for ETVs carried out for secondary hydrocephalus and LOVA, with recurrence of symptoms occurring a number of months or years post-operatively in a small proportion of patients in some series [1, 4, 7–10]. Furthermore, studies have presented patients in whom ETV did not facilitate objective improvement in cognitive or neuropsychological tests, which may reflect the progressive nature of the condition and inter-individual differences in neural compliance after a period of chronic hydrocephalus [4]. In patients with recurrent symptoms repeat ETV or shunt insertion can sometimes deliver symptomatic improvement, but this is again unpredictable. Thus, decisions to re-operate need to be guided by individual patient circumstances. Many cases of LOVA treated with ETV, including those in our series, have demonstrated a benefit in the short-to-medium term. However long-term follow-up, over a period of years, should be considered, given the reports of recurrence and progression of symptoms in a minority of LOVA patients up to 2–3 years post-operatively [4, 10, 13].

Two major advantages of ETV over traditional shunting procedures are the high infection (and thus revision) rates for shunt devices and the risk of shunt over-drainage and its sequale in the latter. After the dramatic complications following PPV insertion in the initial series by Oi et al., focus shifted onto use of gravitational shunts for LOVA patients to minimise the risk of over-drainage [1, 2, 11, 12]. In an important study of shunt procedures for LOVA patients, 23 patients elected gravitational shunt insertion after being offered the choice of ETV or shunt insertion [11, 12]. Two patients (9 %) developed small subdural haematomas, one of which required drainage. Eighty-two percent (19) of the cohort demonstrated clinical improvement after shunt insertion (follow-up 6–75 months). The authors conclude that gravitational shunts are a viable alternative to ETV in the management of LOVA. However, it is of note that 15 % (4) of patients developed symptoms of overdrainge post-operatively, two of which necessitated shunt revision surgery. Median follow-up was 29 months, which may not have been extensive enough to capture cases of later shunt failure or infection in the cohort. Furthermore, the work was limited by the non-randomised methodology for the selection of patients for shunt insertion and the institution’s relatively large, rigid endoscopes leading to strict criteria for excluding ETV as a therapeutic option (foramen of Munro width <6.5 mm and third ventricle floor thickness >2.5 mm).

When discussing treatment options for LOVA, the significant infection and revision rates associated with shunt procedures, and the risks of under- or over-drainage, must be considered [4]. Conversely, the ventricular anatomy may not always be amenable to endoscopic CSF diversion procedures. A comparative study between ETV and shunt implantation for LOVA is yet to be completed; such work could provide useful data to aid therapeutic decision-making for these patients.

## Conclusion

The mysterious pathophysiology underlying LOVA makes therapeutic decision-making for these patients complicated. Unchecked, its progressive nature can lead to morbidity, with poor cognitive, psychological and neurological outcomes [4].

It is vital that patients are counselled about the fact that surgical intervention for LOVA does not bring with it a guarantee of long-term symptomatic relief or neuropsychological improvement. The compliance of brain parenchyma is highly variable between individuals and the capacity for recovery after intervention does not appear to correlate with radiological arrest of hydrocephalus [1, 11, 13].

However, the potential for slowing or halting progression via an established and safe procedure such as ETV makes it an attractive therapeutic option for both clinician and patient. ETV obviates many of the the risks associated with CSF diversion implant procedures, but gravitational shunts may be considered in cases unsuitable for ETV or after recurrence of symptoms after initial ETV [2, 11, 12]. Such recurrence may occur months to years after initial ETV. Long-term follow-up should be implemented after surgery for LOVA.
